# Effects of altering the ratio of C16:0 and *cis*-9 C18:1 in rumen bypass fat on growth performance, lipid metabolism, intestinal barrier, cecal microbiota, and inflammation in fattening bulls

**DOI:** 10.1186/s40104-024-01052-1

**Published:** 2024-07-07

**Authors:** Haixin Bai, Haosheng Zhang, Congwen Wang, Modinat Tolani Lambo, Yang Li, Yonggen Zhang

**Affiliations:** 1 College of Animal Science and Technology, Northeast Agricultural University, Harbin, 150030 China; 2 Faculty of Animal Science and Technology, Yunnan Agricultural University, Kunming, 650500 China

**Keywords:** C16:0, *cis*-9 C18:1, Finishing bulls, Intestinal homeostasis, Lipid metabolism, Low-grade inflammation

## Abstract

**Background:**

C16:0 and *cis*-9 C18:1 may have different effects on animal growth and health due to unique metabolism in vivo. This study was investigated to explore the different effects of altering the ratio of C16:0 and *cis*-9 C18:1 in fat supplements on growth performance, lipid metabolism, intestinal barrier, cecal microbiota, and inflammation in fattening bulls. Thirty finishing Angus bulls (626 ± 69 kg, 21 ± 0.5 months) were divided into 3 treatments according to the randomized block design: (1) control diet without additional fat (CON), (2) CON + 2.5% palmitic acid calcium salt (PA, 90% C16:0), and (3) CON + 2.5% mixed fatty acid calcium salt (MA, 60% C16:0 + 30% *cis*-9 C18:1). The experiment lasted for 104 d, after which all the bulls were slaughtered and sampled for analysis.

**Results:**

MA tended to reduce 0–52 d dry matter intake compared to PA (DMI, *P* = 0.052). Compared with CON and MA, PA significantly increased 0–52 d average daily gain (ADG, *P* = 0.027). PA tended to improve the 0–52 d feed conversion rate compared with CON (FCR, *P* = 0.088). Both PA and MA had no significant effect on 52–104 days of DMI, ADG and FCR (*P* > 0.05). PA tended to improve plasma triglycerides compared with MA (*P* = 0.077), significantly increased plasma cholesterol (*P* = 0.002) and tended to improve subcutaneous adipose weight (*P* = 0.066) when compared with CON and MA. Both PA and MA increased visceral adipose weight compared with CON (*P* = 0.021). Only PA increased the colonization of Rikenellaceae, *Ruminococcus* and Proteobacteria in the cecum, and MA increased *Akkermansia* abundance (*P* < 0.05). Compared with CON, both PA and MA down-regulated the mRNA expression of Claudin-1 in the jejunum (*P* < 0.001), increased plasma diamine oxidase (DAO, *P* < 0.001) and lipopolysaccharide (LPS, *P* = 0.045). Compared with CON and MA, PA down-regulated the *ZO-1* in the jejunum (*P* < 0.001) and increased plasma LPS-binding protein (LBP, *P* < 0.001). Compared with CON, only PA down-regulated the Occludin in the jejunum (*P* = 0.013). Compared with CON, PA and MA significantly up-regulated the expression of *TLR-4* and *NF-κB* in the visceral adipose (*P* < 0.001) and increased plasma IL-6 (*P* < 0.001). Compared with CON, only PA up-regulated the *TNF-α* in the visceral adipose (*P* = 0.01). Compared with CON and MA, PA up-regulated *IL-6* in the visceral adipose (*P* < 0.001), increased plasma TNF-α (*P* < 0.001), and reduced the IgG content in plasma (*P* = 0.035). Compared with CON, PA and MA increased C16:0 in subcutaneous fat and *longissimus dorsi* muscle (*P* < 0.05), while more C16:0 was also deposited by extension and desaturation into C18:0 and *cis*-9 C18:1. However, neither PA nor MA affected the content of *cis*-9 C18:1 in *longissimus dorsi* muscle compared with CON (*P* > 0.05).

**Conclusions:**

MA containing 30% *cis*-9 C18:1 reduced the risk of high C16:0 dietary fat induced subcutaneous fat obesity, adipose tissue and systemic low-grade inflammation by accelerating fatty acid oxidative utilization, improving colonization of *Akkermansia*, reducing intestinal barrier damage, and down-regulating *NF-κB* activation.

## Introduction

The modern view of nutrition is that the animal’s need for fat is actually the need for fatty acids (FA) [1]; therefore, the field of animal nutrition began to focus on the unique effects of individual FA metabolic differences on animal performance [2–4]. C16:0, as the most abundant saturated FA (SFA) in conventional feed materials or animal body fat (including adipose tissue or intramural fat) [5, 6], has been widely used to improve the production performance of animals, especially in dairy cows, due to its efficient energy effect [7]. During periods of positive energy balance, supplementation with C16:0 in dairy cow diets can improve milk production, milk fat production, body tissue reserve and weight gain [4]. However, reports on how purified C16:0 regulates the performance of beef cattle through specific in vivo metabolism are rare. Meanwhile, C16:0 is an SFA, and its studies in monogastric animals have shown that long-term intake poses the risk of excessive obesity and results in lipid metabolism disorders [8]. C16:0 could also reduce the colonization of intestinal anti-inflammatory microbes [9], increase intestinal permeability, increase lipopolysaccharide (LPS) into circulation [10], and then lead to tissue and systemic low-degree inflammation [11–13], and ultimately harm the health of the animal. It is still unclear whether supplementing C16:0 on fattening beef cattle will also cause these adverse effects. In addition, the supplemented SFA can also be easily deposited by animals through ingestion, increasing the content of SFA in animal products such as meat, milk and eggs [14] and causing health concerns. However, there are still few reports on how C16:0-enriched dietary fat affects the FA deposition of beef cattle.

As a monounsaturated FA (MUFA), the anti-inflammatory properties of *cis*-9 C18:1 have been widely revealed in the study of human metabolic syndrome [15]. It can alleviate lipid metabolism disorder and SFA-induced inflammation by accelerating the oxidative utilization of FA [16], reducing intestinal epithelial barrier damage and inhibiting the activation of inflammatory pathway signals [9, 13]. Studies in lactating dairy cows also showed that increasing *cis*-9 C18:1 supplementation could improve the distribution of energy to the body tissue and thus increase the body weight [17]. However, it is unknown how C16:0 and *cis*-9 C18:1 regulate growth and health in finishing cattle through differential metabolism in vivo.

Therefore, we hypothesized that supplementing dietary fat with high C16:0 in fattening cattle would increase their weight gain and fat deposition but, at the same time, could cause body lipid metabolism disorders and inflammation. Partial substitution of C16:0 with *cis*-9 C18:1 can not only maintain the higher performance of fattening cattle but also alleviate or even eliminate the adverse effects of C16:0 on health by regulating intestinal homeostasis and inflammatory pathways. Finally, it will provide some theoretical basis for precision, efficiency, and healthy FA nutrition of beef cattle.

## Materials and methods

### Animals and experimental design

All the procedures of this study were carried out following the guidelines approved by the Animal Ethics Committee of Northeast Agricultural University (Protocol number: NEAUEC2023 02 59). Thirty Angus bulls (21 ± 0.5 months) with an initial body weight of 626 ± 69 kg were assigned to three treatments through a randomized block design, with 10 bulls in each treatment (bulls were blocked by initial weight). The three treatments were: (1) control diet without additional fat (CON), (2) CON + 2.5% palmitic calcium salt (PA, 90% C16:0), (3) CON + 2.5% mixed FA calcium salt (MA, 60% C16:0 + 30% *cis*-9 C18:1). Both fat supplements are rumen bypass fats. Diets were formulated according to the nutritional requirements for this weight stage and the target daily gain of 1.5 kg [18]. The fat addition of the two fat-treated diets is 2.5% of the dietary dry matter (DM), replacing the zeolite powder in the CON diet, and other ingredients were the same. Hence, the three diets had similar crude protein (CP) content, and the PA and MA diets had similar energy content. The composition of diets and fat supplements is shown in Tables 1 and 2. Each bull was fed in a single pen (4 m × 3 m) twice a day at 0800 and 1700, and provided free drinking water. To ensure ad-libitum feeding, the feed amount was adjusted according to the feed intake of the previous day to get about 3% orts. Before the formal trial began, all bulls went through a 14-d transition period to adapt to the new diet and management. In the first 7 d, the test diet replaced 1/3 of the original diet, and in the last 7 d, 2/3 of the original diet, until the start of the test, when it had been completely replaced with the test diet. The formal experiment lasted for 104 d.

**Table 1:** Ingredients and nutrient composition of treatment diets

Item	Diet^1^		
	CON	PA	MA
Ingredient, % of DM			
Corn grain	45.00	45.00	45.00
Soybean meal	8.50	8.50	8.50
Distillers dried grains with solubles	5.00	5.00	5.00
Corn germ meal	5.50	5.50	5.50
Limestone	1.00	1.00	1.00
Salt (sodium chloride)	0.80	0.80	0.80
Sodium bicarbonate	1.10	1.10	1.10
Mineral-vitamin premix^2^	0.40	0.40	0.40
Magnesium oxide	0.20	0.20	0.20
Peanut hulls	15.00	15.00	15.00
Corn stalk	15.00	15.00	15.00
Zeolite powder	2.50	--	--
Palmitic calcium salt	--	2.50	--
Mixed FA calcium salt	--	--	2.50
Nutrient composition, % of DM^3^			
CP	11.56	11.59	11.53
EE	3.74	6.25	6.23
NDF	34.28	34.30	34.28
ADF	20.32	20.36	20.36
Ash	7.97	7.27	7.29
NEm, Mcal/kg	1.76	1.83	1.82
NEg, Mcal/kg	1.15	1.20	1.19
Fatty acids, g/100 g DM			
C16:0	1.47	3.76	2.95
C18:0	0.38	0.65	0.47
* cis*-9 C18:1	1.15	1.18	1.91
* cis*-9,*cis*-12 C18:2	0.46	0.52	0.68

^1^ CON: control diet without additional fat; PA: CON + 2.5% DM palmitic calcium salt (90% C16:0); MA: CON + 2.5% DM mixed FA calcium salt (60% C16:0 + 30% *cis*-9 C18:1)

^2^ The mineral-vitamin premix provided the following per kilogram of the diet: VA 6,000 IU, VD 600 IU, VE 50 IU, Fe 50 mg, Cu 15 mg, Mn 27 mg, Zn 65 mg, Se 0.1 mg, I 0.5 mg, Co 0.2 mg

^3^ *CP* Crude protein, *EE* Ether extract, *NDF* Neutral detergent fiber, *ADF* Acid detergent fiber, *NEm* Net energy for maintenance, *NEg* Net energy for gain. NEm and NEg levels were estimated according to NASEM [18], and the rest of the nutrient levels were analyzed

**Table 2:** Composition of fat supplements (% of total FA)

Item^2^	Fat supplement^1^	
	Palmitic calcium salt	Mixed FA calcium salt
SFA		
C16:0	90.36	60.45
C18:0	6.78	2.93
Total SFA	97.14	63.38
MUFA		
* cis*-9 C18:1	1.62	30.87
PUFA		
* cis*-9,*cis*-12 C18:2	1.07	3.86
Total UFA	2.69	34.73
Other	0.17	1.89

^1^ After the two kinds of fat supplements were designed, Sanhe Feed Co., Ltd. (Harbin, China) was entrusted to produce them

^2^ *FA* Fatty acids, *SFA* Saturated fatty acids, *MUFA* Monounsaturated fatty acids, *PUFA* Polyunsaturated fatty acids, *UFA* Unsaturated fatty acids

### Data and sample collection

#### Body weight, feed and blood

All bulls were weighed before morning feeding on d 0, 52, and 104 to determine average daily gain (ADG). The amount of feed and orts per day were recorded to calculate dry matter intake (DMI). Feed samples were collected once a week and were dried at 55 °C for 48 h, crushed and passed through a 1-mm sieve before storing at 4 °C until nutrient analysis. At 0 d and 104 d, blood was collected from the tail vein of the bull using a heparin sodium tube (Saihua, Heze, China) before morning feeding and then centrifuged at 2,000 × *g* for 15 min at 4 °C to obtain plasma, which was stored at −20 °C until laboratory analysis.

#### Slaughter samples

At the end of the trial, all the bulls were transported to Haosheng abattoir (Harbin, China), 15 km from the fattening farm, for slaughter (fasting for 24 h, but had free access to water). Bulls were electrically stunned and slaughtered by exsanguination, and the skin, head, forefeet, hind feet and viscera were removed. Then, according to the method of Gotoh et al. [19], subcutaneous adipose and visceral adipose (including perirenal, omental, and intestinal adipose) were dissected and weighed. The jejunum tissue, visceral adipose (mesenteric), and cecal contents were collected with a cryostorage tube, frozen with liquid nitrogen and placed in a −80 °C refrigerator until q-PCR analysis and microbial sequencing were performed. *Longissimus dorsi* muscle between the 12^th^ and 13^th^ ribs and subcutaneous adipose tissue were stored at −20 °C for intramuscular fat content and FA composition analysis.

### Laboratory analysis

#### Feed nutrients, muscle fat content, and FA profile

As described in AOAC [20], feed DM was analyzed by method 934.01, ether extract (EE) by method 954.05, CP by method 968.06, ash by method 942.05, and muscle fat content was determined by method 920.29. The neutral detergent fiber (NDF) and acid detergent fiber (ADF) in feed were analyzed by heat-stable α-amylase treatment according to the method of van Soest et al. [21].

The FA profile was analyzed according to the method of Choi et al. [22]. Firstly, feed samples and fat supplements were acidified with 3 mol/L HCl solution. Then, total lipids in 5 g of feed, 100 mg of fat supplements, 100 mg subcutaneous adipose, and 100 mg of *longissimus dorsi* muscle were extracted respectively with chloroform-methanol (2:1, v/v). Then, the FA methyl esterification (FAME) was carried out according to the description of ISO 5009 [23]. Using the nonadecanoic acid (C19:0) as the internal standard. The FAME was determined by a gas chromatography-flame ionization detector (GC-2010 Plus autoinjector-AOC 20i; Shimadzu Scientific Instruments, Kyoto, Japan) equipped with a fused silica column (SP-2560, 100 m × 0.25 mm i.d. with 0.2-µm film thickness; Supelco Inc., Bellefonte, PA, USA). The initial temperature was 100 °C for 13 min, then it was heated to 180 °C at the rate of 10 °C/min for 6 min, then to 200 °C at the rate of 1 °C/min for 20 min, and finally to 230 °C at the rate of 4 °C/min for 10.5 min. Nitrogen was the carrier gas, the split ratio was 100:1, and the injection volume was 1 µL. The FA profile of each sample was confirmed by comparing the retention time with the standards (FAME mix of 37 components from Supelco Inc., Bellefont, PA, USA).

#### Plasma indices

The plasma samples were sent to the Sino-uk Institute of Biological Technology (Beijing, China) to determine triglycerides (TG) and cholesterol (CHOL) using an automatic biochemical analyzer (Beckmann Coulter AU680, California, USA). The immunoglobulin G (IgG), immunoglobulin M (IgM), immunoglobulin A (IgA), tumour necrosis factor-α (TNF-α), interleukin-6 (IL-6) and interleukin-1β (IL-1β) were determined according to the standard operation of ELISA kit (Sino-uk Institute of Biological Technology, Beijing, China). The diamine oxidase (DAO) activity, D-lactic acid, LPS, and lipopolysaccharide-binding protein (LBP) in plasma were determined using an ELISA kit purchased from Jingmei Biotechnology Co., Ltd. (Jiangsu, China).

#### Relative mRNA expression

The detailed steps for the relative mRNA expression were reported in a previous study by Welboren et al. [24]. In brief, total RNA was extracted separately from 100 mg jejunum and 100 mg visceral adipose using Trizol Reagent (Invitrogen, Carlsbad, CA, USA). The concentration and purity of the extracted RNA were determined using a Nanodrop 2000 ultramicro spectrophotometer (Thermo, America). When the OD_260/280_ value was 1.8–2.0, it indicated that the extracted RNA could be used for subsequent trials. Using the PrimeScript™ RT Reagent Kit (TaKaRa, Dalian, China) and following the manufacturer’s instructions, the extracted total RNA was reverse-transcribed into cDNA. Based on the target gene (Table 3) sequence in GenBank, primers were designed using Primer Premier 6.0 and synthesized by entrusting Sangon Biotech Co., Ltd. (Shanghai, China). The sequence of the tested genes is shown in Table 3. The instructions of SYBR^®^ Premix Ex Taq II (Tli RNaseH Plus) regents (TaKaRa, Dalian, China) were followed strictly to prepare the q-PCR reaction system and the reaction was performed in a CFX-96 Real-Time PCR Detection System (BioRad, USA). The reaction procedures were as follows: initial denaturation at 95 °C for 10 s, followed by 40 cycles of 95 °C for 5 s, 60 °C for 30 s, and 72 °C for 15 s. The steps of the melting curve were as follows: one cycle of 95 °C for 10 s, followed by a change in temperature from 65 °C to 95 °C with a velocity of 0.5 °C/s. β-actin was used as an internal reference gene. The target gene mRNA expression level was calculated according to the 2^−ΔΔCt^ method, where ΔΔCt _(sample−control)_ = (Ct of target gene − Ct of β-actin) _sample_ − (Ct of target genes − Ct of β-actin) _control_.

**Table 3:** Gut permeability and inflammatory gene primer sequences used in q-PCR

Gnenes^1^	Primers	Sequences (5´ to 3´)	Product size, bp	GenBank accession No.
*ZO-1*	F	GAGCCCCCTAGTGATGTGTG	85	XM_024982009.1
	R	GGTTTTAGGATCACAGTGTGGTAG		
Occludin	F	AGTTTCAGGTGAATGGGTCACG	91	NM_001082433.2
	R	CCGCCTGAAGAAGCAGAAAGG		
Claudin-1	F	CCATCTATGAGGGGCTGTGG	107	NM_001001854.2
	R	GGTTGCTTGCAAAGTGCTGT		
Tricellulin	F	CCGCCACTACCTTCGGC	136	XM_005221445.4
	R	GGTGCCATCTGGATAGTCCG		
E-cadherin	F	TTCCCGCCATCCTGGGGATCC	126	AB037667.1
	R	GGGTGTCATCTTCTGGGGGCAGT		
*TNF-α*	F	CCGCATTGCAGTCTCCTACC	110	NM_173966.2
	R	TGGGTTCATACCAGGGCTTG		
*IL-1β*	F	TCCACGTGGGCTGAATAACC	93	NM_174093.1
	R	TCGGGCATGGATCAGACAAC		
*IL-6*	F	CAGGAACGAAAGAGAGCTCCA	87	NM_173923.2
	R	GGGAGACAGCGAATGGAGTG		
*IL-10*	F	TGATGCCACAGGCTGAGAAC	126	NM_174088.1
	R	TCTTGTTTTCGCAGGGCAGA		
*MCP-1*	F	ATCCTCTCGCTGCAACATGAA	127	NM_174006.2
	R	TGTATAGCAGCAGGCGACTT		
*PPARγ*	F	GTGAAGCCCATTGAGGACAT	148	NM_181024
	R	AGCTGCACGTGTTCTGTCAC		
*NF-κB*	F	AAGAGAAGATGGGGAAAGGCTG	249	XM_005226181.1
	R	CGTCGGCAAATGAGAAGTAGTG		
*TLR-4*	F	CGAAAGCAGAAAGCCACAGTT	116	NM_174198.6
	R	AAGCTGGCTTTTATCCCAGGAA		
β-actin	F	AGCAAGCAGGAGTACGATGAGT	120	NM_173979
	R	ATCCAACCGACTGCTGTCA		

^1^ *ZO-1* Zonula occludens-1, *TNF-α *Tumour necrosis factor-α, *IL-1β *Interleukin-1β, *IL-6* Interleukin-6, *IL-10* Interleukin-10, *MCP-1* Monocyte chemoattractant protein-1, *PPARγ* Peroxisome proliferator-activated receptor γ, *NF-κB* Nuclear factor kappa B, *TLR-4* Toll-like receptor 4

#### Cecal microbial composition

The E.Z.N.A.^®^ Stool DNA kit (D4015, Omega, Inc., USA) was used to extract the DNA of cecal contents according to the instructions. The total DNA extracted was eluted with 50 µL elution buffer and stored at −80 °C. Using the extracted DNA as a template, primers (338F 5´-ACTCCTACGGGAGGCAGCA-3´; 806R 5´-GGACTACHVGGGTWTCTAAT-3´) were designed to amplify the 16S rDNA V3–V4 region of the cecal microbiota. All reactions were performed in a 25 µL mixture consisting of extracted template DNA (approximately 25 ng), PCR premixes (12.5 µL), primers (2.5 µL each), and PCR-grade water was added to fill volumes. The procedure of PCR amplification was as follows: predenaturation lasted for 30 s at 98 °C; denaturation lasted for 10 s at 98 °C; annealing at 52 °C for 30 s, elongation at 72 °C for 45 s (35 cycles), and the extension lasted for 10 min at 72 °C. The PCR products were inspected by 2% agarose gel electrophoresis. Ultrapure water was used instead of sample solution as a negative control to rule out the possibility of false positive PCR results. The products of PCR were normalized with AxyPrep^TM^ Mag PCR Normalizer (Axygen Biosciences, Union City, CA, USA). AMPure XT beads (Beckman Coulter Genomics, Danvers, MA, USA) were used to purify and construct the library, and the size and quantity of the library were evaluated by LabChip GX (Perkin Elmer, Waltham, MA, USA) and Library Quantification Kit for Illumina (Kapa Biosystems, Woburn, MA, USA). The constructed library was sequenced by Illumina MiSeq 2 × 300 bp BP double-ended sequencing platform.

FLASH (v1.2.7) was used to splice the original sequencing data, and using FQTRIM (v 0.94) and Vsearch (v 2.3.4) for quality control, chimera filtering, and the operational taxonomic units (OTU) were obtained accordingly. The obtained OTU was used for α-diversity analysis, and QIIME2 was used to calculate Chao1 to evaluate the microbiota abundance. QIIME2 was also used for β-diversity analysis. Using the “ggplot 2” package of R software (v3.3.1) to generate the principal coordinate analysis (PCoA) based on the Bray-Curtis distance matrix. LEfSe analysis (LDA > 3.8) was used to identify the intestinal flora with significant differences between groups.

### Statistical analysis

Data analysis was performed using SAS 9.4 (SAS Inst. Inc., Cary, NC, USA). Since this study was a randomized block design, a mixed model considering the fat treatment as a fixed effect and the weight block as a random effect was applied in the MIXED procedure as follows:

$$Y_{\mathit i\mathit j}=\mu+T_{\mathit i}+B_{\mathit j}+E_{\mathit i\mathit j}$$

Where* Y*_*ij*_ is the observation of bulls that belong to the *j*^th^ weight block and offered a diet containing the *i*^th^ fat supplement, *µ* is the general mean, *T*_*i*_ is the fat supplement effect, *B*_*j*_ is the effect of the weight block, and *E*_*ij*_ represents random residual error. Plasma parameters were also analyzed using a MIXED procedure with d 0 data as a covariate. The least squares means were calculated and separated using the default approach (PDIFF). Differences between fat diets were detected using Duncan’s adjustment. Differences in the cecal microbial composition were analyzed by the Kruskal-Wallis rank sum test with the Tukey–Kramer post hoc test and Benjamini-Hochberg false discovery rate. Declared significant at *P* < 0.05, and trends were declared at 0.05 ≤ *P* < 0.10.

## Results

### Growth performance and obesity phenotype

There was no difference in the initial weight of the bulls in the three treatment groups (*P* = 0.987). In the early stage of the experiment (0–52 d), the MA tended to reduce DMI compared to the PA (*P* = 0.052, Table 4). Compared with CON and MA, the PA significantly increased the ADG (0–52 d) of fattening bulls (*P* = 0.027). Compared with CON, PA tended to improve feed conversion rate (FCR, *P* = 0.088), while MA had no significant effect on FCR (*P* > 0.05). In addition, the DMI, ADG and FCR in the later period of the experiment (52–104 d) were not affected by fat supplementation (*P* > 0.05). Fat supplementation (including PA and MA) had no significant effect on live weight at slaughter (*P* = 0.395, Table 5) and intramuscular fat content of *longissimus dorsi* muscle (*P* = 0.10) but significantly increased visceral adipose weight (*P* = 0.021). Compared with CON and MA, PA significantly increased plasma CHOL (*P* = 0.002), tended to increase subcutaneous adipose weight (*P* = 0.066). PA tended to increase plasma TG (*P* = 0.077) compared with MA.

**Table 4:** Effects of dietary fat with different FA on growth performance of finishing bulls

Item^2^	Treatment^1^			SEM	*P*-value
	CON	PA	MA		
Initial weight, kg	627.12	623.68	628.69	22.194	0.987
0–52 d					
DMI, kg/d	12.28^ab^	12.73^a^	12.15^b^	0.167	0.052
ADG, kg/d	1.28^b^	1.67^a^	1.37^b^	0.099	0.027
FCR (gain:feed intake)	0.10^b^	0.13^a^	0.11^ab^	0.008	0.088
52–104 d					
DMI, kg/d	11.79	12.49	12.05	0.223	0.104
ADG, kg/d	1.16	1.48	1.42	0.081	0.110
FCR (gain:feed intake)	0.10	0.12	0.12	0.009	0.283

^a,b^ Within a row, values with different superscripts differ significantly at *P* < 0.05, and a trend toward at *P* < 0.1

^1^ CON: control diet without additional fat; PA: CON + 2.5% DM palmitic calcium salt (90% C16:0); MA: CON + 2.5% DM mixed FA calcium salt (60% C16:0 + 30% *cis*-9 C18:1). *n* = 10

^2^ *DMI* Dry matter intake, *ADG *Average daily gain, *FCR *Feed conversion rate

**Table 5:** Effects of dietary fat with different FA on obesity phenotype of finishing bulls

Item^2^	Treatment^1^			SEM	*P*-value
	CON	PA	MA		
Live weight at slaughter, kg	753.70	773.75	787.40	17.284	0.395
Subcutaneous adipose, kg	22.71^b^	23.53^a^	22.74^b^	0.270	0.066
Visceral adipose, kg	39.17^b^	41.05^a^	40.73^a^	0.476	0.021
Intramuscular adipose, %	6.81	7.40	7.39	0.218	0.112
TG, mmol/L	0.23^ab^	0.24^a^	0.22^b^	0.006	0.077
CHOL, mmol/L	3.97^b^	4.40^a^	3.94^b^	0.081	0.002

^a,b^ Within a row, values with different superscripts differ significantly at *P* < 0.05, and a trend toward at *P* < 0.1

^1^ CON: control diet without additional fat; PA: CON + 2.5% DM palmitic calcium salt (90% C16:0); MA: CON + 2.5% DM mixed FA calcium salt (60% C16:0 + 30% *cis*-9 C18:1). *n* = 10

^2^*TG* Triglycerides, *CHOL* Cholesterol

### Cecal microbial structure and community

**Fig. 1:**
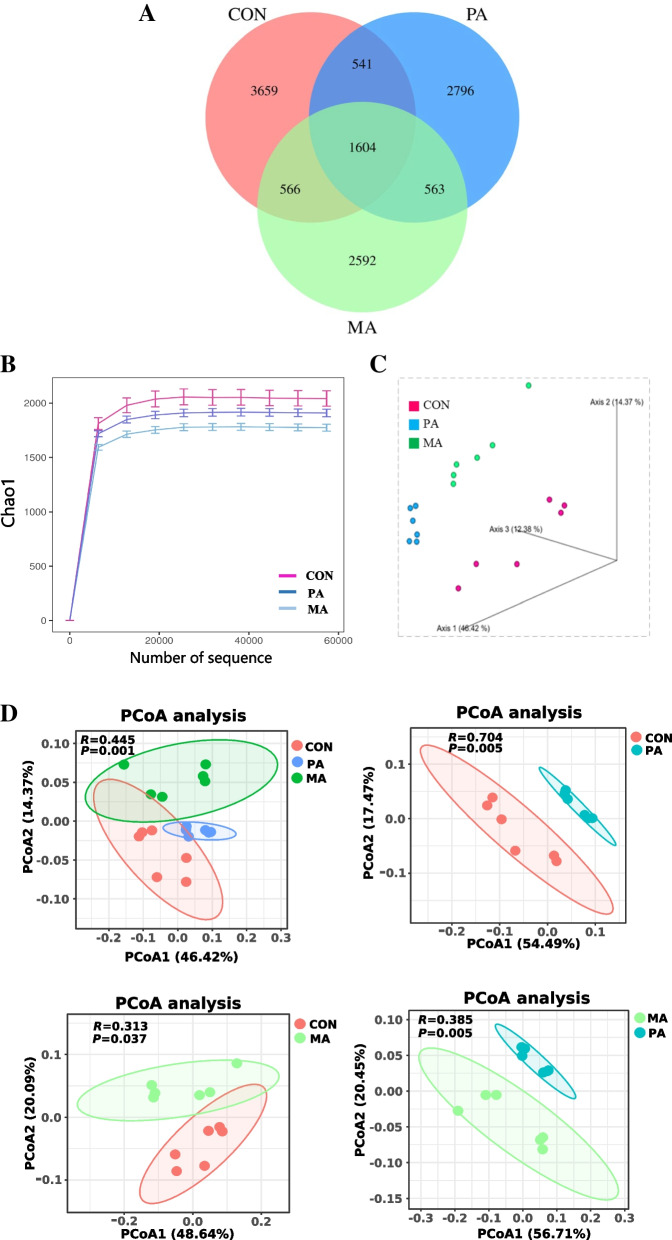
Effects of dietary fat with different FA on α-diversity and β-diversity of cecal microbiota of finishing bulls (*n* = 6). **A** OTU Venn; **B** Rarefaction curve-Chao1; **C** β-diversity-PCoA analysis (3D); **D** β-diversity-PCoA analysis (2D). CON: control diet without additional fat; PA: CON + 2.5% DM palmitic calcium salt (90% C16:0); MA: CON + 2.5% DM mixed FA calcium salt (60% C16:0 + 30% *cis*-9 C18:1)

**Fig. 2:**
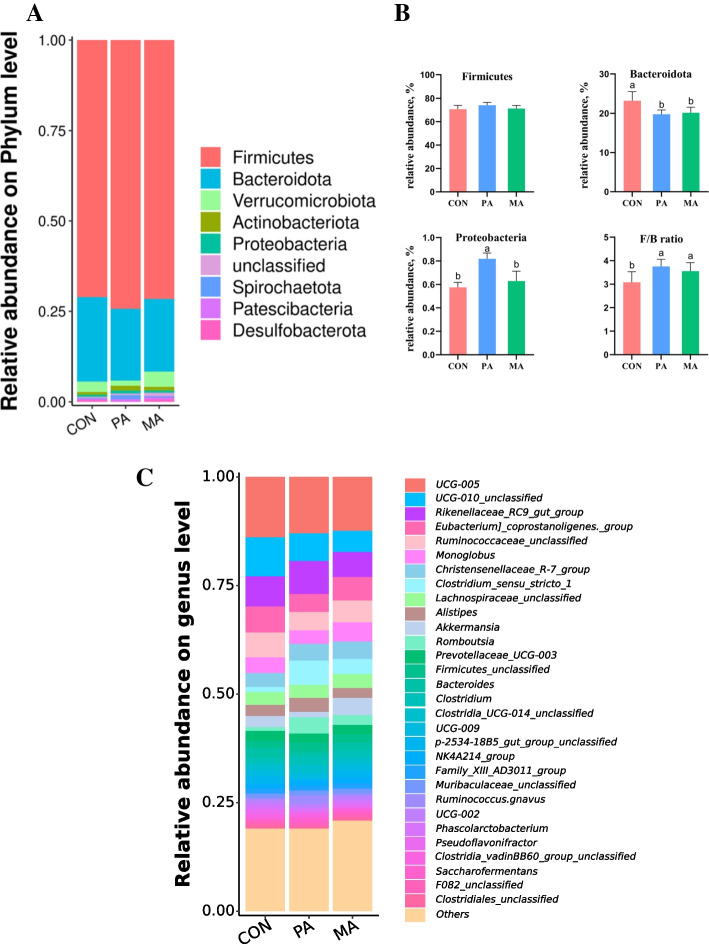
Effects of dietary fat with different FA on cecal microbiota composition at phylum and genus level of finishing bulls (*n* = 6). **A** The composition of microbiota at the phylum level; **B** The differences of microbiota at the phylum level; **C** Microbiota composition at the genus level. CON: control diet without additional fat; PA: CON + 2.5% DM palmitic calcium salt (90% C16:0); MA: CON + 2.5% DM mixed FA calcium salt (60% C16:0 + 30% *cis*-9 C18:1). ^a,b^ Bars with different letters differ significantly at *P* < 0.05. Data are expressed as means ± SEM

**Fig. 3:**
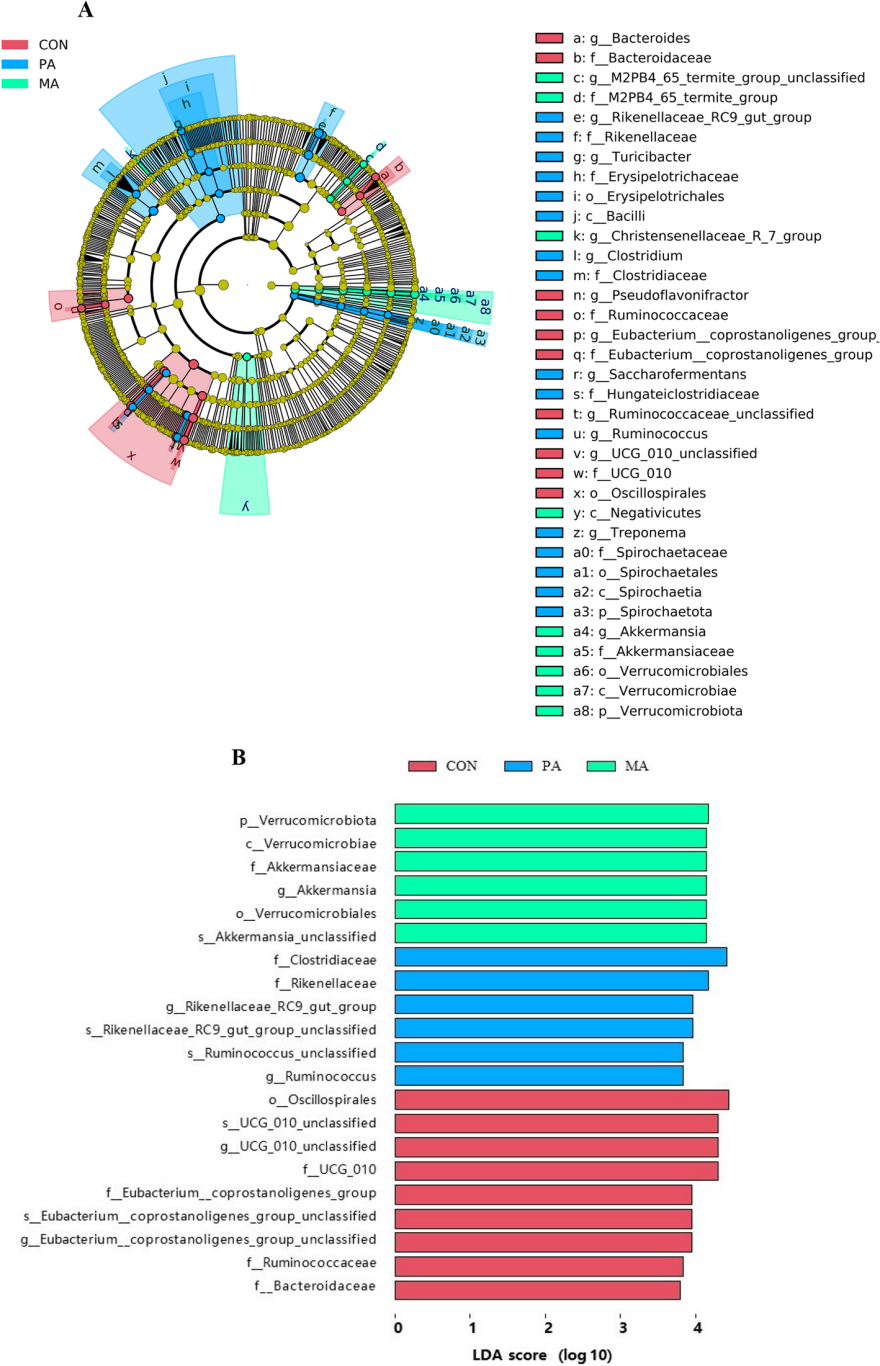
Dietary fat with different FA differentially regulated cecal microbiota in beef cattle (*n* = 6). **A** Cladogram of LEfSe shows taxonomic profiling from the phylum to genus level, the yellow node represents no difference, but other color nodes represent significant difference; **B** Linear discriminant analysis (LDA) distribution, and the score > 3.8 means significant. CON: control diet without additional fat; PA: CON + 2.5% DM palmitic calcium salt (90% C16:0); MA: CON + 2.5% DM mixed FA calcium salt (60% C16:0 + 30% *cis*-9 C18:1)

The raw sequencing reads of cecal microorganisms were obtained by 16S rDNA sequencing. After preprocessing and removing the low-quality sequence, there were more than 70,000 clean reads, and more than 80% were high-quality sequences in the effective sequences of each sample, indicating that the sequencing depth was sufficient to meet the requirements of subsequent analysis. OTU clustering was performed on the obtained high-quality sequence valid tags according to 97% similarity. As shown in Fig. 1A, 3,659, 2,796 and 2,592 unique OTU were identified in the CON, PA and MA, respectively. The richness of cecal microorganisms was reduced by fat-supplemental diets (PA and MA), indicated by decreased Chao1, and the MA decreased more obviously than PA (Fig. 1B). As shown in Fig. 1C, PCoA (3-dimensional) of the weighted UniFrac demonstrated an evident clustering of samples according to diet. The PCoA (2-dimensional) analysis also revealed that the CON, PA, and MA groups formed three distinct clusters with a significant separation trend (Fig. 1D, *P* < 0.05). Firmicutes, Bacteroidota and Verrucomicrobiota, were the dominant phyla in the cecum of bulls under the three diets, accounting for more than 95% (Fig. 2A). Kruskal-Wallis rank sum test was used to determine further the differences of phyla under different diets (Fig. 2B). The 2 fat supplement diets (PA and MA) did not affect the relative abundance of Firmicutes (*P* = 0.116), but increased the ratio of Firmicutes to Bacteroidota (F/B ratio, *P* = 0.021) by reducing Bacteroidota (*P* = 0.006). In addition, only the PA significantly increased the abundance of Proteobacteria (*P* < 0.001). At the genus level (Fig. 2C), the predominant genera were *Ruminococcaceae_UCG-005, Ruminococcaceae_UCG-010_unclassified*, *Rikenellaceae_RC9_gut_group*, *Eubacterium_coprostanoligenes_group*, and so on. LEfSe analysis (LDA > 3.8) was used to screen the signature intestinal microbial flora under the fat supplementation, and the results of the evolutionary tree were shown in Fig. 3A. Our findings identified 21 distinct bacterial taxa across three diets (Fig. 3B). Of these, 9 bacterial taxa were specific to the CON, 6 were specific to the PA, and 6 were specific to the MA. Clostridiaceae, Rikenellaceae, *Rikenellaceae_RC9_gut_group*, *Rikenellaceae_RC9_gut_group_unclassified, Ruminococcus*, *Ruminococcus_unclassified* under the PA were detected. Verrucomicrobiota, Verrucomicrobiae, Verrucomicrobiales, Akkermansiaceae, *Akkermansia*, *Akkermansia_unclassified* under the MA were detected.

### Gut permeability and endotoxemia

**Fig. 4:**
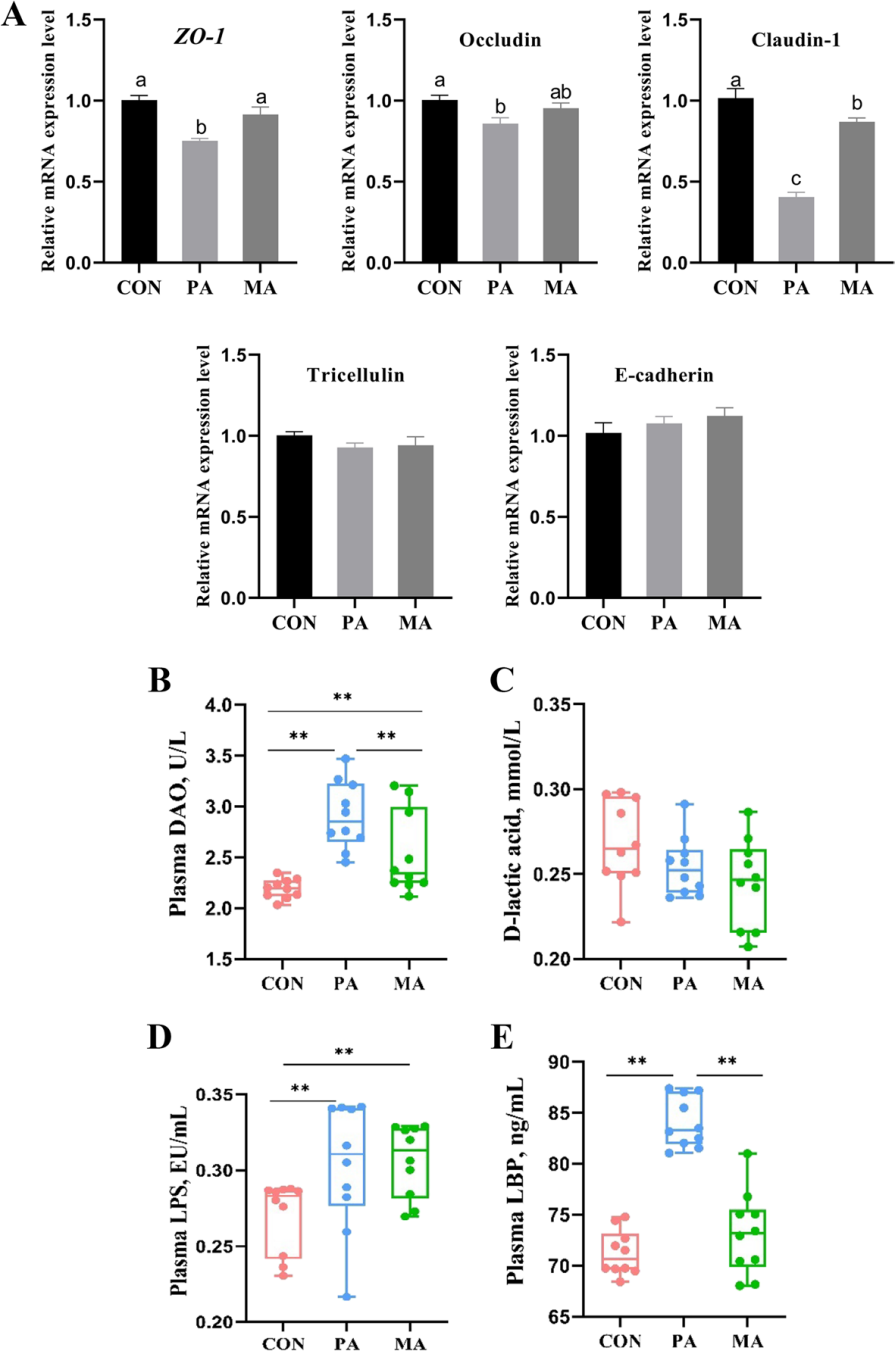
Effects of dietary fat with different FA on gut permeability and endotoxemia of finishing bulls (*n* = 10). **A** The relative mRNA expression of zonula occludens-1 (*ZO-1*), Occludin, Claudin-1, Tricellulin, E-cadherin; **B** Plasma diamine oxidase (DAO); **C** Plasma D-lactic acid; **D** Plasma lipopolysaccharide (LPS); **E** Plasma lipopolysaccharide-binding protein (LBP). CON: control diet without additional fat; PA: CON + 2.5% DM palmitic calcium salt (90% C16:0); MA: CON + 2.5% DM mixed FA calcium salt (60% C16:0 + 30% *cis*-9 C18:1).^a,b^ Bars with different letters differ significantly at *P *< 0.05, **Representing *P* < 0.05 in the box plot. Data are expressed as means ± SEM

As shown in Fig. 4A, compared with CON, the PA significantly down-regulated the mRNA expression of zonula occludens-1 (*ZO-1*, *P* < 0.001) and Occludin (*P* = 0.013) genes in the jejunum of fattening bulls, but the MA had no significant effect (*P* > 0.05). Both fat supplementation (PA and MA) significantly down-regulated the expression of Claudin-1, and the expression level of PA was also significantly lower than that of MA (*P* < 0.001). In addition, fat supplementation had no significant effect on the expression of Tricellulin and E-cadherin (*P* > 0.05). Two types of fat supplementation (PA and MA) significantly increased the content of intestinal permeability marker plasma DAO, and MA was lower than PA (Fig. 4B, *P* < 0.001), but the other marker D-lactic acid was not affected (Fig. 4C, *P* = 0.197). Both fat supplementation significantly increased plasma LPS content (Fig. 4D, *P* = 0.045). Only PA fat increased plasma LBP (Fig. 4E, *P* < 0.001).

### Plasma inflammatory factors and immunoglobulins

As shown in Table 6, compared with CON and MA, the PA significantly increased the content of TNF-α in the plasma of finishing bulls (*P* < 0.001). Both fat supplement diets (PA and MA) significantly increased plasma IL-6 concentration compared with CON, but the magnitude of the increase in MA was lower than that in PA (*P* < 0.001). In addition, fat supplementation had no significant effect on plasma IL-1β (*P* = 0.748). Compared with CON and MA, the PA significantly reduced the IgG content in plasma (*P* = 0.035). Both PA and MA had no significant effect on IgA (*P* = 0.459) and IgM (*P* = 0.241).

**Table 6:** Effects of dietary fat with different FA on plasma inflammatory factors and immunoglobulins of finishing bulls

Item	Treatment^1^			SEM	*P*-value
	CON	PA	MA		
Inflammatory factors, pg/mL					
TNF-α	54.18^b^	63.79^a^	55.51^b^	0.865	< 0.001
IL-6	118.27^c^	144.48^a^	128.74^b^	1.837	< 0.001
IL-1β	25.56	25.04	25.40	0.343	0.748
Immunoglobulins, g/L					
IgA	1.68	1.76	1.75	0.047	0.459
IgG	8.40^a^	7.58^b^	8.25^a^	0.206	0.035
IgM	1.20	1.15	1.24	0.027	0.241

^a–c^ Means within rows with different superscripts differ (*P* < 0.05)

^1^ CON: control diet without additional fat; PA: CON + 2.5% DM palmitic calcium salt (90% C16:0); MA: CON + 2.5% DM mixed FA calcium salt (60% C16:0 + 30% *cis*-9 C18:1). *n* = 10

### Expression of inflammatory response genes in visceral adipose

**Fig. 5:**
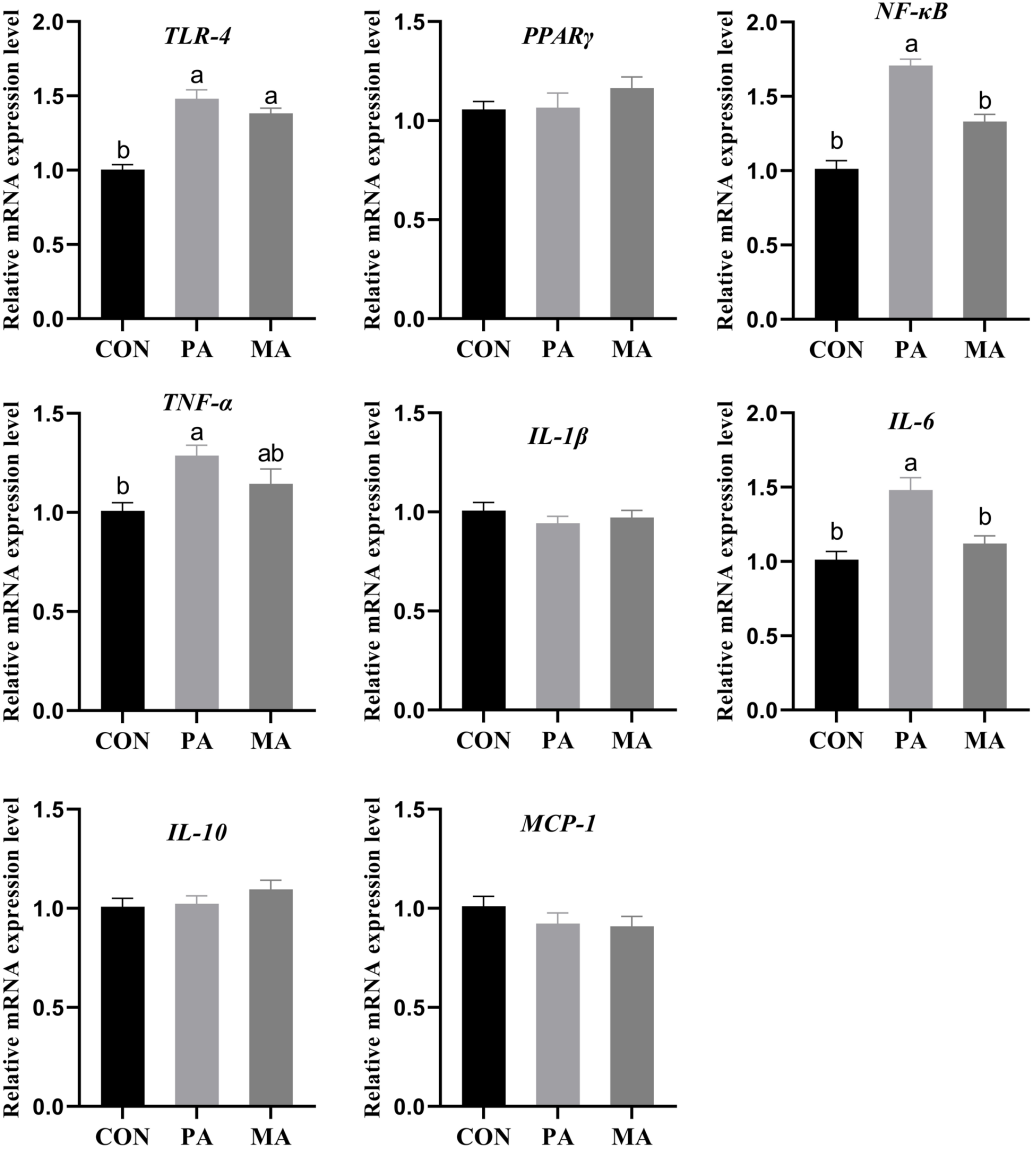
Effects of dietary fat with different FA on mRNA expression of inflammatory response gene in visceral adipose of finishing bulls (*n* = 10). TLR-4: Toll-like receptor 4; PPARγ: Peroxisome proliferator-activated receptor γ; NF-κB: Nuclear factor kappa B; TNF-α: Tumour necrosis factor-α; IL-1β: Interleukin-1β; IL-6: Interleukin-6; IL-10: Interleukin-10; MCP-1: Monocyte chemoattractant protein-1. CON: control diet without additional fat; PA: CON + 2.5% DM palmitic calcium salt (90% C16:0); MA: CON + 2.5% DM mixed FA calcium salt (60% C16:0 + 30% *cis*-9 C18:1). ^a,b^ Bars with different letters differ significantly at *P* < 0.05. Data are expressed as means ± SEM

As shown in Fig. 5, compared with CON, both fat supplementation diets (PA and MA) significantly up-regulated the expression of toll-like receptor 4 (*TLR-4*, *P* < 0.001) and nuclear factor kappa B (*NF-κB*, *P* < 0.001) in the visceral adipose tissue, and the *NF-κB* expression of MA was lower than that of PA. Only PA significantly up-regulated *TNF-α* (*P* = 0.01) and *IL-6* (*P* < 0.001), and the *TNF-α* expression of MA was not significant compared with CON and MA. In addition, fat supplementation had no significant effect on the expression of *IL-1β*, *IL-10*, peroxisome proliferator-activated receptor γ (*PPARγ*), monocyte chemoattractant protein-1 (*MCP-1*) in visceral adipose tissue (*P* > 0.05).

### FA profile of subcutaneous adipose and *longissimus dorsi* muscle

Table 7 shows the FA composition of subcutaneous adipose in fattening bulls. Both fat supplement diets (PA and MA) significantly increased the contents of C16:0 (*P* = 0.007), C17:0 (*P* = 0.003), total FA (ΣFA, *P* < 0.001), and tended to increase *cis*-9 C18:1 (*P* = 0.056). Compared with CON and MA, the PA significantly increased the content of C18:0 (*P* < 0.001) and total saturated FA (ΣSFA, *P* = 0.001) but significantly reduced the content of C21:0 (*P* < 0.001). Compared with CON, PA reduced the ratio of monounsaturated FA (MUFA) to SFA (MUFA/SFA, *P* = 0.026) and unsaturated FA to SFA (UFA/SFA, *P* = 0.029) in subcutaneous adipose, but there was no significant difference in MA compared to CON and PA (*P* > 0.05).

**Table 7:** Effects of dietary fat with different FA on the FA profile (% of total FA) of subcutaneous adipose of finishing bulls

Item	Treatment^1^			SEM	*P*-value
	CON	PA	MA		
C14:0	2.95	3.08	2.94	0.112	0.649
C14:1	1.80	1.67	1.58	0.076	0.130
C15:0	0.26	0.25	0.27	0.011	0.345
C16:0	25.9^b^	28.39^a^	27.39^a^	0.508	0.007
C16:1	8.57	8.12	8.29	0.220	0.359
C17:0	0.51^b^	0.59^a^	0.61^a^	0.020	0.003
C17:1	0.57	0.54	0.55	0.018	0.548
C18:0	6.84^b^	8.09^a^	7.10^b^	0.155	< 0.001
*cis*-9 C18:1	44.59^b^	45.86^a^	45.74^a^	0.391	0.056
C18:2 n-6c	1.65	1.58	1.71	0.080	0.520
C18:2 n-6t	0.14	0.14	0.13	0.003	0.807
C18:3 n-3	0.28	0.26	0.27	0.010	0.611
C21:0	0.83^a^	0.68^b^	0.79^a^	0.025	< 0.001
Σ SFA^2^	37.28^b^	41.08^a^	39.11^b^	0.642	0.001
Σ UFA^3^	57.60	58.17	58.28	0.492	0.580
Σ MUFA^4^	55.53	56.19	56.16	0.482	0.559
Σ PUFA^5^	2.07	1.99	2.12	0.083	0.524
Σ n-3 FA^6^	0.28	0.26	0.27	0.010	0.659
Σ n-6 FA^7^	1.80	1.73	1.86	0.079	0.523
Σ FA^8^	94.89^b^	99.25^a^	97.39^a^	0.693	< 0.001
n-6/n-3 FA	6.50	6.80	6.95	0.338	0.639
MUFA/SFA	1.49^a^	1.37^b^	1.44^ab^	0.029	0.026
PUFA/SFA	0.06	0.05	0.05	0.003	0.346
UFA/SFA	1.55^a^	1.42^b^	1.49^ab^	0.031	0.029

^a,b^ Within a row, values with different superscripts differ significantly at *P* < 0.05, and a trend toward at *P* < 0.1

Fatty acids detected at < 0.1% of total fatty acid are not reported

^1^ CON: control diet without additional fat; PA: CON + 2.5% DM palmitic calcium salt (90% C16:0); MA: CON + 2.5% DM mixed FA calcium salt (60% C16:0 + 30% *cis*-9 C18:1). *n* = 10

^2^ Sum of saturated fatty acids

^3^ Sum of unsaturated fatty acids

^4^ Sum of monounsaturated fatty acids

^5^ Sum of polyunsaturated fatty acids

^6^ Sum of n-3 polyunsaturated fatty acids

^7^ Sum of n-6 polyunsaturated fatty acids

^8^ Sum of fatty acids

Table 8 shows the composition of fatty acids in the *longissimus dorsi* muscle of finishing bulls. In general, FA composition in beef is greatly affected by fat supplementation. Compared with CON, PA increased C14:0, C18:3 n-3, and total n-3 FA (Σn-3 FA) content, but MA reduced them (*P* < 0.05). MA increased n-6/n-3 FA compared with CON and PA (*P* < 0.001). Both fat supplement diets (PA and MA) significantly increased C16:0 and C18:0 content (*P* < 0.001) and reduced the content of C16:1 (*P* < 0.001). However, the other FA of focus, *cis*-9 C18:1, did not show significant differences in beef under the three diets (*P* > 0.05). The total polyunsaturated FA (ΣPUFA) content of the two fat supplement diets did not differ compared with CON (*P* > 0.05), but PA reduced ΣPUFA compared with MA (*P* = 0.042). Compared with CON and MA, the PA significantly increased *cis*-9 C20:1, ΣSFA and ΣFA (*P* < 0.05), while significantly decreased C18:2 n-6, total UFA (ΣUFA), total MUFA (ΣMUFA), total n-6 FA (Σn-6 FA), MUFA/SFA, UFA/SFA (*P* < 0.05).

**Table 8:** Effects of dietary fat with different FA on the FA profile (% of total FA) of *longissimus dorsi* muscle of finishing bulls

Item	Treatment^1^			SEM	*P*-value
	CON	PA	MA		
C12:0	0.16	0.15	0.16	0.006	0.984
C14:0	2.21^b^	2.48^a^	2.02^c^	0.028	< 0.001
C15:0	0.25	0.25	0.23	0.007	0.105
C16:0	28.22^b^	29.09^a^	28.05^a^	0.151	< 0.001
C16:1	3.79^a^	2.49^c^	3.43^b^	0.111	< 0.001
C17:0	0.65	0.66	0.65	0.019	0.975
C17:1	0.60	0.61	0.56	0.022	0.266
C18:0	16.51^c^	18.62^a^	17.21^b^	0.185	< 0.001
*cis*-9 C18:1	41.03	40.99	41.37	0.364	0.723
C18:2 n-6c	2.34^a^	2.12^b^	2.40^a^	0.064	0.011
C18:3 n-3	0.79^b^	0.84^a^	0.72^c^	0.016	< 0.001
*cis*-9 C20:1	0.10^b^	0.13^a^	0.10^b^	0.004	< 0.001
C20:3 n-6	0.16	0.15	0.16	0.006	0.182
C20:4 n-6	1.03	1.02	1.09	0.054	0.613
Σ SFA^2^	48.01^b^	51.26^a^	48.32^b^	0.225	< 0.001
Σ UFA^3^	49.83^a^	48.34^b^	49.82^a^	0.417	0.026
Σ MUFA^4^	45.52^a^	44.22^b^	45.45^a^	0.403	0.053
Σ PUFA^5^	4.32^ab^	4.13^b^	4.37^a^	0.068	0.042
Σ n-3 FA^6^	0.79^b^	0.84^a^	0.72^c^	0.016	< 0.001
Σ n-6 FA^7^	3.53^a^	3.29^b^	3.65^a^	0.072	0.005
Σ FA^8^	97.84^b^	99.61^a^	98.14^b^	0.403	0.010
n-6/n-3 FA	4.50^b^	3.94^c^	5.14^a^	0.158	< 0.001
MUFA/SFA	0.95^a^	0.86^b^	0.94^a^	0.011	< 0.001
PUFA/SFA	0.09	0.08	0.09	0.001	0.134
UFA/SFA	1.04^a^	0.94^b^	1.03^a^	0.011	< 0.001

^a–c^ Within a row, values with different superscripts differ significantly at *P* < 0.05, and a trend toward at *P* < 0.1

Fatty acids detected at < 0.1% of total fatty acid are not reported

^1^ CON: control diet without additional fat; PA: CON + 2.5% DM palmitic calcium salt (90% C16:0); MA: CON + 2.5% DM mixed FA calcium salt (60% C16:0 + 30% *cis*-9 C18:1). *n* = 10

^2^ Sum of saturated fatty acids

^3^ Sum of unsaturated fatty acids

^4^ Sum of monounsaturated fatty acids

^5^ Sum of polyunsaturated fatty acids

^6^ Sum of n-3 polyunsaturated fatty acids

^7^ Sum of n-6 polyunsaturated fatty acids

^8^ Sum of fatty acids

## Discussion

### Growth performance and obesity phenotype

Previous studies in lactating cows have shown that DMI is mainly affected by the amount of fat supplements and their FA composition [25], but there is no consensus. When supplemented with fat rich in C16:0, C18:0 and *cis*-9 C18:1 at 1.7%–5% of dietary DM content, dairy cows showed no effect [26], increase or decrease [27, 28] in DMI. In this study, fat addition was equal, so the observed 0–52 d DMI of MA lower than PA is likely due to differences in FA composition. *cis*-9 C18:1 has been shown to promote the secretion of gut peptides related to satiety (i.e., cholecystokinin and glucagon-like peptide 1) [29], thereby delaying the rate of gastrointestinal emptying and ultimately decreasing DMI. This may be the reason why *cis*-9 C18:1-enriched MA reduces DMI in bulls. Compared with CON, PA increased 0–52 d ADG, possibly due to the higher dietary energy concentration after fat supplementation. However, PA improved the 0–52 d ADG compared to MA, possibly due to its higher DMI, because 60%–90% of animal performance can be attributed to DMI [30]. Furthermore, the prominent ADG of PA also brought about a higher FCR.

The lipid metabolism of beef cattle in the late stage of fattening is crucial for metabolic homeostasis, weight gain, and meat quality, as fat accounts for over 55% of weight gain during the fattening period of about 500 kg [18]. It has traditionally been assumed that all dietary fats enhance animal performance primarily by exerting energy effects. However, recent studies have focused more on individual FA differences in metabolism because they can have unique effects on animal performance [31]. Specifically, compared to SFA such as C16:0, UFA *cis*-9 C18:1 is preferentially mobilized, and as a fuel source, its oxidation energy supply rate is faster, thus reducing weight gain or having a tendency to inhibit fat growth [32]. This may be another reason why MA’s ADG was lower than PA in the 0–52 d. Intramuscular and intermuscular are ideal fat deposit sites for fattening cattle, which can improve meat quality and growth performance [33]. When a large amount of fat is deposited in the subcutaneous or visceral tissues, more “waste fat” will be produced [19], which easily leads to “ectopic obesity”. Compared with CON, PA with higher dietary energy increased the weight of subcutaneous and visceral adipose in bulls, possibly due to the easier fattening of C16:0 [34]. Although the differential metabolism between *cis*-9 C18:1 and C16:0 did not affect the chief fat depots visceral adipose (PA and MA’s visceral adipose weight did not differ), it reversed the increase in MA’s subcutaneous adipose, as MA subcutaneous adipose weight was lower than PA. In addition, the content of *cis*-9 C18:1 in subcutaneous fat was the highest compared with visceral fat and intramuscular fat [35], so it could be mobilized and utilized to the highest extent. This may be the reason why the differential metabolism of FA only affects the subcutaneous fat.

Compared with CON and MA, C16:0-enriched PA elevated plasma CHOL, and PA also increased plasma TG compared with MA, which increased the risk of lipid metabolism disorders in bulls. According to the report by De Souza et al. [36], compared to C18:0, *cis*-9 C18:1, and *cis*-9,*cis*-12 C18:2, C16:0 was more capable of stimulating de novo synthesis of FA and incorporating into TG. In the blood, CHOL is transmitted mainly in two forms: high-density lipoprotein cholesterol (HDL-C) and low-density lipoprotein cholesterol (LDL-C). High-SFA (C16:0) diets are capable of boosting CHOL synthesis and lowering the affinity of LDL to LDL receptors [37]. LDL receptors are the main receptors for clearing circulating LDL-C. Therefore high C16:0 diet ultimately leads to an increase in CHOL levels in the plasma. MA containing 30% *cis*-9 C18:1 did not result in this adverse effect, which means that *cis*-9 C18:1 may be more effective in maintaining lipid metabolism homeostasis due to special metabolic mechanisms, although the relevant mechanisms remain to be elucidated. Studies in skeletal muscle cells and mice have shown that C16:0 up-regulates CHOL synthesis [38], while *cis*-9 C18:1 inhibits TG secretion [15], which is consistent with our results.

### Cecal microbial structure and community

In order to avoid the hydrogenation of dietary FA by rumen microorganisms and reduce the harmful effects of UFA on rumen microorganisms, the fat supplements used in this study were made into FA calcium salts, which were typical rumen bypass fat [39]. Fatty acid calcium salts dissolved in the lower pH of the abomasum and small intestine and reappeared as non-esterified FA [4, 40, 41]. Whether the non-esterified FA interfere with the gut microbes in the same way they do with the rumen microbes is unknown. At the same time, studies have shown that the imbalance of intestinal flora caused by dietary fat can affect the host’s lipid metabolism and the development of systemic inflammation [42]. We therefore performed 16S rDNA sequencing on cecal microbes to reveal these effects of dietary FA. Studies have shown that dietary FA intake affects gut microbiota diversity, and most of the time unfavourably [43, 44], consistent with our result because, compared with CON, the Chao1 index, i.e., microbiota richness, decreased after fat supplementation. In addition, the significant difference in the PCoA index among PA, MA, and CON also indicated that dietary fat significantly alters the microbial community structure of finishing bulls.

As the two most dominant bacteria at the phylum level, over 90% in this study, Firmicutes and Bacteroidota play a crucial role in intestinal energy metabolism and the development of obesity [45]. In mice research, a higher F/B ratio in the gut has been linked to obesity [46]. Compared with CON, the two fat supplements in this study increased F/B, meaning there was a higher energy utilization to promote weight gain in bulls. In humans and mice, obesity is usually accompanied by a decrease in Bacteroidota [47], which may correspond to significantly lower levels of Bacteroidota observed in PA and MA compared to CON. However, contrary findings in mice concluded that consuming C16:0-enriched dietary fat increases the colonization of Bacteroidota [9, 48]. Therefore, further research is needed to determine whether this difference resulted from the difference in experimental animals. The low-grade inflammation observed in obese individuals has been attributed to the immune response to increased circulating levels of LPS from the Gram-negative bacterial cell wall [49]. All Proteobacteria were Gram-negative bacteria [50]. Therefore, it was observed that C16:0-enriched PA increased Proteobacteria abundance and likely increased intestinal LPS to induce tissue inflammation in obese bulls.

Further, at the genus level, *Ruminococcaceae_UCG-005* and *Ruminococcaceae_UCG-010_unclassified* were the most dominant genus in the cecum of cattle, which was consistent with the study of He et al. [51] on fattening cattle. The LEfSe analysis results showed that PA increased the abundance of Rikenellaceae, *Rikenellaceae_RC9_gut_group*, *Rikenellaceae_RC9_gut_group_unclassified*, *Ruminococcus*, *Ruminococcus_unclassified*, which were believed to regulate energy metabolism and promoted lipid generation [52, 53]. These microbial communities might mediate high C16:0 for higher fat deposition and weight gain. *Akkermansia*, a subgroup of Verrucomicrobiota, was significantly up-regulated in MA. *Akkermansia* has significant anti-inflammatory properties, and it has been shown to be negatively associated with obesity phenotypes such as adipose tissue inflammation and dyslipidemia [54–56]. UFA, especially n-3 FA intake, can increase the colonization of *Akkermansia*, thereby improving the integrity of the intestinal epithelial barrier and exerting anti-inflammatory effects [57]. Although there is no specific study on the impact of *cis*-9 C18:1 on *Akkermansia*, *cis*-9 C18:1 in MA is highly likely to ameliorate excess obesity and subsequent tissue inflammation in bulls through this mechanism.

In summary, C16:0 enriched-PA promoted the fattening of bulls and caused potential intestinal barrier damage by increasing the colonization of Rikenellaceae, *Ruminococcus* and Proteobacteria in the cecum. MA containing 30% *cis*-9 C18:1 alleviated excessive obesity and intestinal barrier damage induced by C16:0 by increasing *Akkermansia* abundance.

### Gut permeability and endotoxemia

Studies of long-term fat supplementation in rodents have shown that tissue inflammation and metabolic disorders are associated with increased intestinal permeability [58, 59]. The endotoxemia, i.e., the passage of Gram-negative bacterial cell wall LPS into the systemic circulation, is a consequence of an altered intestinal barrier and is the main contributor to the low-grade inflammation triggered by dietary fat-induced excessive obesity [49]. All of these emphasize the importance of the intestinal epithelial barrier in lipid challenge-induced inflammation. Although perturbations of the gut microbiota caused by dietary lipids can partially mediate changes in the gut barrier, lipids themselves can also increase intestinal permeability by directly affecting tight junctions [60]. Here, we demonstrated that supplementation of C16:0, *cis*-9 C18:1-enriched dietary fats (PA and MA) was sufficient to disrupt the intestinal barrier of the bull jejunum by directly down-regulating the expression of Claudin-1, a key part of the tight junction. This defect in the intestinal barrier was consistent with increased levels of plasma DAO of PA and MA, a marker of intestinal permeability [61]. However, further observation showed that in comparison to CON, high C16:0 PA may exacerbate the deterioration of intestinal permeability by significantly down-regulating the other two key genes of tight junction *ZO-1* and Occludin. Studies on Caco-2/TC7 cells have shown that C16:0 can lead to increased paracellular permeability by directly disrupting intercellular connections [9]. However, *cis*-9 C18:1 has no effect on the integrity of the intestinal barrier and can even counteract or diminish the harmful effects of C16:0 on cellular function [15]. This may partly explain why 30% *cis*-9 C18:1 + 60% C16:0 MA has less severe intestinal barrier damage than PA.

A high-fat diet facilitates the colonization of Gram-negative bacteria in the gut [62]. In particular, SFA increased the abundance of Proteobacteria (Gram-negative staining) in the gut, as discussed above [63]. All of these factors increase the production of intestinal LPS. A compromised intestinal barrier due to fat diets, especially high-C16:0 PA diet, may exacerbate LPS penetration. Non-esterified FA absorbed through the epithelium of the small intestine is re-esterified to form chylomicrons together with lipoproteins [4, 64]. Chylomicrons readily bind to LPS and carry them into the blood in large quantities [65]. Therefore, we observed that in comparison to CON, long-term supplementation of either high-saturated C16:0 fat or 30% *cis*-9 C18:1 + 60% C16:0 fat increased LPS in the blood of bulls. This may be a direct response to chronic lipid load independent of dietary FA saturation. The secretion of LBP, which mediates endotoxin inflammation [66], was found to be elevated in the PA compared to the CON. This increase was likely a response to higher levels of LPS. However, the lower LBP in MA compared to PA may be due to the presence of *cis*-9 C18:1, as it can inhibit the expression and release of LBP, thereby weakening the development of inflammation [67]. Overall, chronic lipid challenges increased intestinal permeability and LPS entry in fattening bulls independently of dietary lipid saturation. However, the presence of *cis*-9 C18:1 mitigated the damage of C16:0 to the intestinal barrier to some extent.

### Adipose tissue and systemic low-grade inflammation

Dietary SFA and LPS may share the same inflammatory signalling pathway, thereby enhancing the development of inflammation [68]. Mesenteric adipose is directly in contact with the intestine, and dietary FA enters the blood through mesenteric adipose’s lymphatic vessels [69], so visceral fat represented by the mesenteric fat will bear greater metabolic pressure and also tend to cause more complex and stronger inflammatory responses [70]. mRNA expression analysis of inflammation response genes in mesenteric fat showed that *TLR-4* was up-regulated regardless of FA type. *TLR-4* is the connection hub between the consumption of dietary fats and metabolic inflammation [10]. LPS is a natural ligand of *TLR-4* [71], and changes in gut microbiota and permeability caused by PA and MA increase the entry of LPS, which may strongly activate *TLR-4*. In addition, SFA can act as non-microbial agonists of *TLR-4* [72], so C16:0-enriched PA may also directly activate the expression of *TLR-4* compared to the CON. Activation of *TLR-4* can promote the expression of pro-inflammatory transcription factors, such as *NF-κB*, thereby stimulating the transcription of inflammatory cytokines [68]. However, the presence of *cis*-9 C18:1 in MA did not cause the promotion of *NF-κB* expression as strongly as in PA. This is consistent with several other studies in skeletal muscle cells, vascular endothelial cells, and adipose tissue, suggesting that *cis*-9 C18:1 can reduce the inflammatory effect of C16:0 by reducing the activation of *NF-κB* [11–13]. PA’s inflammatory cytokines *TNF-α* and *IL-6* may be stimulated by *NF-κB*, and their expression is increased, which means that C16:0-enriched PA may bring a more intense inflammatory response in visceral adipose tissues. Actually, changes in gut microbiota induced by lipid metabolism, increased gut permeability, endotoxemia, tissue, and systemic inflammation are complexly interrelated occurrences in obesity [73]. Obesity caused by dietary fat, especially SFA C16:0, may eventually cause systemic low-grade inflammation by disrupting these processes [9]. As we observed in the results, compared with CON and MA, C16:0-enrich PA resulted in the highest levels of pro-inflammatory cytokines TNF-α and IL-6 in plasma. At the same time, IgG levels decreased, suggesting that chronic low-grade inflammation associated with obesity has contributed to the weakened immunity of finishing bulls. Overall, the presence of *cis*-9 C18:1 reduces C16:0 and LPS-induced adipose tissue inflammation by reducing the activation of *NF-κB*, thereby reducing the development of systemic inflammation.

### FA profile of subcutaneous adipose and *longissimus dorsi* muscle

Fatty acids in ruminant meat and milk have two main origins: direct dietary intake or de novo synthesis using acetic acid [74]. Direct intake accounts for about 40%–60% of total fat [75]. For FA in milk fat, the medium and short chain FA (< 14 C) mainly depends on de novo synthesis. As for C16:0, half of it is synthesized de novo, while the other half needs to be ingested. Almost all of the 18 C FA (including C18:0, *cis*-9 C18:1, *cis*-9,*cis*-12 C18:2, C18:3 n-3) rely on direct dietary intake [76]. These results strongly suggest that dietary supplementation of fats rich in C16:0, C18:0, and *cis*-9 C18:1 is sufficient to influence the FA composition of ruminants’ products by direct absorption. So it is not surprising that PA and MA significantly increased the C16:0 content of subcutaneous fat and *longissimus dorsi* muscle compared to the CON. At the same time, the sharp increase of C16:0 also pulled down the UFA/SFA in meat and subcutaneous adipose. This could raise health concerns, as intake of SFA C16:0 is often linked to metabolic diseases such as atherosclerosis and type 2 diabetes [15, 77]. But it was also noticed that PA increased C18:0 in meat and fat compared to the CON and MA. It may be derived from a large extension of C16:0 [4]. This is because both ingested and synthesized FA are subject to the action of elongase and desaturase [78]. Although C18:0 is SFA, it is harmless to cardiovascular disease and even healthy [75]. C18:0 could be heavily desaturated by stearoyl CoA desaturase (SCD) to *cis*-9 C18:1, and *cis*-9 C18:1 is healthy. It was reported that with the increase of adipose tissue of fattening cattle, the expression and catalytic activity of the SCD enzyme gene will increase sharply [79]. In beef cattle from weaning to 16 months old, the MUFA/SFA ratio in meat increased from 0.66 to 0.86, mainly because SCD catalyzed the production of more *cis*-9 C18:1 [79]. In addition, SFA C16:0 and C18:0 strongly promoted the activation of SCD [80]. Therefore, *cis*-9 C18:1 of MA in subcutaneous fat was higher than CON in our results, which may be partly due to ingestion and partly due to lengthening and desaturation of C16:0. While PA’s *cis*-9 C18:1 is also higher than CON, it is more likely to be achieved by massively lengthening and desaturating C16:0 to *cis*-9 C18:1. However, there was no observed difference in *cis*-9 C18:1 content in *longissimus dorsi* muscle. This may be because the activity of the SCD enzyme in subcutaneous fat is twice that of intramuscular fat [35, 79], so the difference in *cis*-9 C18:1 in subcutaneous fat is greater, and the content is higher than in meat. Overall, supplementation of C16:0-enriched dietary fat increased SFA C16:0 in subcutaneous fat and *longissimus dorsi* muscle, while more C16:0 was also deposited by extension and desaturation into C18:0 and *cis*-9 C18:1 can properly balance the higher SFA and alleviate health concerns to some extent.

## Conclusion

High C16:0 fat supplement improved the 0–52 d growth performance of fattening bulls. Replacing C16:0 with 30% *cis*-9, C18:1 reduced the risk of subcutaneous fat obesity, adipose tissue and systemic low-grade inflammation by accelerating FA oxidative utilization, improving colonization of anti-inflammatory bacteria *Akkermansia*, reducing intestinal barrier damage, and down-regulating *NF-κB* activation. However, the presence of *cis*-9 C18:1 did not improve the *cis*-9 C18:1 in beef and also pulled down the fattening effect of C16:0 to some extent. Further research is needed to determine the optimal ratio of C16:0 and *cis*-9 C18:1 for both efficient weight gain and physically healthy maintenance of finishing cattle.

## Data Availability

All datasets generated for this research are available from the first and corresponding authors upon reasonable request.

## Abbreviations

ADG: Average daily gain
CHOL: Cholesterol
CP: Crude protein
DAO: Diamine oxidase
DM: Dry matter
DMI: Dry matter intake
FA: Fatty acid
FAME: Fatty acid methyl esterification
F/B: Firmicutes/Bacteroidota
FCR: Feed conversion rate
IgA: Immunoglobulin A
IgG: Immunoglobulin G
IgM: Immunoglobulin M
IL-1β: Interleukin-1β
IL-6: Interleukin-6
IL-10: Interleukin-10
LBP: LPS-binding protein
LPS: Lipopolysaccharide
MUFA: Monounsaturated fatty acid
NF-κB: Nuclear factor kappa B
OUT: Operational taxonomic units
PUFA: Polyunsaturated fatty acid
SFA: Saturated fatty acid
TG: Triglycerides
TLR-4: Toll-like receptor 4
TNF-α: Tumour necrosis factor-α
ZO-1: Zonula occludens-1
